# Event-Trigger Reinforcement Learning-Based Coordinate Control of Modular Unmanned System via Nonzero-Sum Game

**DOI:** 10.3390/s25020314

**Published:** 2025-01-07

**Authors:** Yebao Liu, Tianjiao An, Jianguo Chen, Luyang Zhong, Yuhan Qian

**Affiliations:** 1 Aerospace Times Feihong Technology Company Limited, Beijing 130012, China; liu-yebao@outlook.com (Y.L.); chen-jian-guo@outlook.com (J.C.); zhong-luyang@outlook.com (L.Z.); qian-yuhan@outlook.com (Y.Q.); 2 Department of Control Science and Engineering, Changchun University of Technology, Changchun 130012, China

**Keywords:** reinforcement learning, nonzero-sum game, optimal control, event-trigger

## Abstract

Decreasing the position error and control torque is important for the coordinate control of a modular unmanned system with less communication burden between the sensor and the actuator. Therefore, this paper proposes event-trigger reinforcement learning (ETRL)-based coordinate control of a modular unmanned system (MUS) via the nonzero-sum game (NZSG) strategy. The dynamic model of the MUS is established via joint torque feedback (JTF) technology. Based on the NZSG strategy, the existing coordinate control problem is transformed into an RL issue. With the help of the ET mechanism, the periodic communication mechanism of the system is avoided. The ET-critic neural network (NN) is used to approximate the performance index function, thus obtaining the ETRL coordinate control policy. The stability of the closed-loop system is verified via Lyapunov’s theorem. Experiment results demonstrate the validity of the proposed method. The experimental results show that the proposed method reduces the position error by 30% and control torque by 10% compared with the existing control methods.

## 1. Introduction

With the rapid development of the space industry and the continuous increase in the demand for space exploration, the complexity of the environment and the precision of the control requirements faced by space operations are also constantly improving [1,2,3]. The problems of high risk and low efficiency caused by traditional astronaut operations relying on them leaving the capsule are becoming increasingly prominent. In recent years, thanks to the rapid improvement and development of unmanned systems research and development technology, the use of high-precision and -performance unmanned systems to solve the assembly and maintenance of space operations in orbit is gradually becoming a scientific value of the goal-oriented basic research topic in the field of space exploration. So far, unmanned systems operating in conventional ground environments have achieved good reliability and accuracy. However, under the high standard requirements of coordinate missions in complex space environments, traditional unmanned systems are difficult to meet the transportation requirements of launch vehicles and spacecraft due to their large volume, heavy mass, and difficulty in disassembly and assembly. However, for the space unmanned systems that are in service to overcome the above difficulties, there are still some limitations in the configuration of the mechanism, and it is difficult to change its assembly configuration and working mode according to different task requirements. The modular unmanned system (MUS) [4,5] is a kind of autonomous unmanned system with standard modules and interfaces that can reassemble and configure itself according to different task requirements. Through the reconfiguration of modules, the unmanned system can show a variety of assembly configurations to complete different tasks, thus showing advantages that traditional unmanned systems do not possess.

As an important branch of game theory, differential game [6,7] focuses on the dynamic decision-making process of continuous time systems described by differential equations. It is an ideal tool to deal with multi-participant decision making and control problems and to solve optimal strategies, and it is widely used in economics [8], management [9], computer science [10], and other fields [11,12]. Reinforcement learning [13,14] originated as an imitation of the human brain learning mechanism, reflecting the mapping of learning environment state to action, so that the system can obtain the maximum cumulative reward from the environment and then optimize the system performance through the optimal strategy selection. In recent years, it has been widely used in complex nonlinear differential games because it can effectively solve the problem of “dimensionality disaster” in traditional dynamic programming [15,16]. As a kind of game, the nonzero-sum game (NZSG) [17,18] needs to solve the corresponding coupled Hamilton–Jacobi (HJ) equation for each player in order to obtain its Nash equilibrium solution.

As an important part of modern control theory, the core problem of optimal control is to select control strategies to make some performance indexes of a given controlled system optimal. For a large number of nonlinear systems in practical engineering, to obtain the optimal control strategy, it is necessary to solve the HJ(-Bellman) (HJB) equation, which is a class of nonlinear partial differential equation, and it is difficult to obtain the optimal solution by analytical methods. The reinforcement learning method is a powerful tool to solve the optimal control problem of nonlinear systems. In reinforcement learning systems, a neural network (NN) [19,20] is designed to approximate the performance index function and estimate the solution of the HJ(B) equation. Due to its strong advantages in solving nonlinear optimal control, reinforcement learning has attracted extensive attention from scholars both domestic and abroad in recent years, and has made rich achievements in solving problems such as discrete time optimal control [21,22,23], continuous time optimal control [24,25,26], and data-driven optimal control [27,28,29] of complex nonlinear systems. However, these results are based on periodic sampling or event triggering, resulting in a waste of resources and high computational costs.

Motivated by the above, this paper develops event-trigger (ET) reinforcement learning (ETRL)-based coordinate control of MUS via the NZSG strategy. The main contributions of this paper are mainly the following two aspects:

1. To the best of the authors’ knowledge, it is the first time to introduce the NZSG via reinforcement learning applied to an MUS. By considering the control torque of *n* modules in the MUS as decision-makers, the optimal control problem for the MUS system is morphed into an NZSG issue with *n* players.

2. The stability of the developed method is guaranteed and the experiment on MUS is conducted. Through the experimental results, we can conclude that the proposed method produces less tracking errors and power consumption.

## 2. Background and Related Work

### 2.1. Reinforcement Learning

Optimal control is widely utilized and holds great importance in many areas. However, with the increase in the system’s dimension, the issue of the dimensionality curse has appeared. Reinforcement learning, as an effective solution to the dimensionality curse in optimal control, has emerged as a crucial approach for addressing approximate optimal control issues. Vamvoudakis et al. [30] published a book about RL-based control for cognitive autonomy. Wang et al. [12] developed a review in the field of RL for advanced control applications. Liu and Xue et al. [31] concluded the RL with applications in control. The above three papers are all surveys about the RL or adaptive dynamic programming. The proposed method in this paper deals with the coordinate control of an modular unmanned system that is a specific application environment using RL. Dong et al. [32] proposed safe RL for the sake of trajectory tracking for a modular robot system. Event-trigger is not mentioned, as it causes computation and communication burdens. An et al. [17] designed a cooperative game-based RL method for human–robot interaction. Liu et al. [33] used the RL control method to deal with the vehicle path tracking issue. The particle swarm optimization method is utilized in the vehicle path tracking issue [34]. However, the above methods only consider a single controller to guarantee optimality. The modern industry needs more than one player/controller to finish the task using other player’s information. Hence, the developed nonzero-sum game has great importance.

### 2.2. Nonzero-Sum Game

Differential game theory focuses on the dynamic decision-making process in multi-player interactive systems and with advantages in dealing with uncertain interaction and disturbance. Differential games include the zero-sum game, nonzero-sum game, cooperative game, etc. Each module in the MUS system functions as a participant in NZSG, each with its own policy, collectively operating within the group using a general quadratic performance index function as the basis for the game. Wu et al. [35] proposed NZSG for an unmanned aerial vehicle with uncertain as well as asymmetric information. Coordinate control is not considered. Zheng et al. [36] developed a Q-learning-based NZSG for spacecraft system under a pursuit–evasion condition. The above method is based on a time-triggered mechanism. Besides optimum, the communication burden between the sensor and actuator needs to be considered. Therefore, an event-trigger has been developed to decrease the quantity of sampling. An et al. [37] used a dynamic event-trigger to complete a robot’s tracking task via NZSG. Dong et al. [38] proposed event-trigger value iteration RL for a coordinated task under the framework of NZSG. The above methods are only applied on robots; thus, they are unsuitable for modular unmanned systems.

## 3. Dynamic Model

For an MUS employing the JTF technique, the dynamic model of the *i*th subsystem is presented below:

(1) $$I_{im}\gamma_{i}{\ddot{q}}_{i}+\frac{\tau_{is}}{\gamma_{i}}+f_{ir}\left(q_{i},{\dot{q}}_{i}\right)+I_{i}\left(q,\dot{q},\ddot{q}\right)=\tau_{i}+J_{i}^{T}f,$$

where *f* is the contact force between the MUS and object; $f_{ir}\left(q_{i},{\dot{q}}_{i}\right)$ means lumped joint friction; $\gamma_{i}$ indicates the gear ratio; $q_{i}$ reflects joint position; $\tau_{is}$ represents coupled joint torque; $I_{i}\left(q,\dot{q},\ddot{q}\right)$ is the IDC effect among MUS subsystems; $\tau_{i}$ indicates control torque; and subscript *i* is *i*th joint module subsystem. The property analyses are described below:

(1) The lumped joint friction

The joint friction term $f_{ir}\left(q_{i},{\dot{q}}_{i}\right)$ is formulated as

(2) $$\begin{array}{cc} f_{ir}\left(q_{i},{\dot{q}}_{i}\right) & ={\hat{f}}_{ib}{\dot{q}}_{i}+\left({\hat{f}}_{is}e^{\left(-{\hat{f}}_{ir}{\dot{q}}_{i}^{2}\right)}+{\hat{f}}_{ic}\right)sgn\left({\dot{q}}_{i}\right)+f_{ip}\left(q_{i},{\dot{q}}_{i}\right)+Y_{i}\left({\dot{q}}_{i}\right){\tilde{F}}_{ir}, \end{array}$$

in which

(3) $$Y_{i}\left({\dot{q}}_{i}\right)={\left[f_{ib}-{\hat{f}}_{ib},f_{ic}-{\hat{f}}_{ic},f_{is}-{\hat{f}}_{is},f_{i\tau }-{\hat{f}}_{i\tau }\right]}^{T},$$

where $f_{ip}\left(q_{i},{\dot{q}}_{i}\right)$ is the position dependency friction term; $f_{ib},f_{i\tau }$ are viscous and Stribect friction effects; and $f_{is},f_{ic}$ are static and Coulomb friction parameters. Furthermore, ${\hat{f}}_{ib},{\hat{f}}_{ic},$ ${\hat{f}}_{is},$ and ${\hat{f}}_{i\tau }$ are the estimated values.

**Remark** **1.** *The variables $f_{ib},f_{ic},f_{is},f_{i\tau }$ are bounded, and their corresponding estimates also possess boundedness. Consequently, this ensures that the variable ${\tilde{F}}_{ir}$ is bounded, as indicated by $\left|{\tilde{F}}_{ir}\right|\leq b_{iFrm}$, where $b_{iFrm}$ represents a known positive constant for each m in (1,2,3,4). Consequently, $Y_{i}\left({\dot{q}}_{i}\right){\tilde{F}}_{ir}$ can be derived, which is designated as $\left|Y_{i}\left({\dot{q}}_{i}\right){\tilde{F}}_{ir}\right|\leq Y_{i}\left({\dot{q}}_{i}\right)b_{iFrm}$. Additionally, $\left|f_{ip}\left(q_{i},{\dot{q}}_{i}\right)\right|\leq b_{iFp}$, in which $b_{iFp}$ is a known positive constant.*

(2) The interconnected dynamic coupling

The IDC is expressible as a nonlinear function of the coupled vectors of the entire modular subsystem in this way:

(4) $$\begin{array}{cc} I_{i} & =I_{im}\sum_{j=1}^{i-1}v_{mi}^{T}v_{lj}{\ddot{q}}_{j}+I_{im}\sum_{j=2}^{i-1}\sum_{k=1}^{j-1}v_{mi}^{T}\left(v_{lk}\times v_{lj}\right){\dot{q}}_{k}{\dot{q}}_{j}\\ \; & =I_{im}\sum_{j=1}^{i-1}D_{j}^{i}{\ddot{q}}_{j}+I_{im}\sum_{j=2}^{i-1}\sum_{k=1}^{j-1}\Theta_{kj}^{i}{\dot{q}}_{k}{\dot{q}}_{j}\\ \; & =\sum_{j=1}^{i-1}\left[I_{im}{\hat{D}}_{j}^{i},I_{im}\right]{\left[{\ddot{q}}_{j},{\tilde{D}}_{j}^{i}{\ddot{q}}_{j}\right]}^{T}+\sum_{j=2}^{i-1}\sum_{k=1}^{j-1}\left[I_{im}{\hat{\Theta }}_{kj}^{i},I_{im}\right]{\left[{\ddot{q}}_{j},{\tilde{\Theta }}_{kj}^{i}{\dot{q}}_{k}{\dot{q}}_{j}\right]}^{T}, \end{array}$$

in which $v_{mi},v_{lj},v_{lk}$ denote the unit vectors along with the *i*th, *j*th, and *k*th joint rotation axes, respectively. Consequently, define $D_{j}^{i}=v_{mi}^{T}v_{lj}$ and $\Theta_{kj}^{i}=v_{mi}^{T}\left(v_{lk}\times v_{lj}\right)$. We also have the relation that ${\hat{D}}_{j}^{i}=D_{j}^{i}-{\tilde{D}}_{j}^{i}$ and ${\hat{\Theta }}_{kj}^{i}=\Theta_{kj}^{i}-{\tilde{\Theta }}_{kj}^{i}$, in which ${\hat{D}}_{j}^{i},{\hat{\Theta }}_{kj}^{i}$ represent the estimated values of $D_{j}^{i},\Theta_{kj}^{i}$, and ${\tilde{D}}_{j}^{i},{\tilde{\Theta }}_{kj}^{i}$ are alignment errors.

**Remark** **2.** *Based on (4), which characterizes $v_{mi},v_{lk},v_{lj}$, it is inferred that the magnitudes of the associated vector products are bounded, where $\left|D_{j}^{i}\right|=\left|v_{mi}^{T}v_{lj}\right|<1$ and $\left|\Theta_{kj}^{i}\right|=\left|v_{mi}^{T}\left(v_{lk}\times v_{lj}\right)\right|<1$. Additionally, our findings indicate that $I_{i}$ is bounded and the up-bound is given as $\left|I_{i}\right|\leq b_{iI}$ with a positive constant.*

Define state vector $x_{i}={\left[x_{i1},x_{i2}\right]}^{T}={\left[q_{i},{\dot{q}}_{i}\right]}^{T}$ and the control input $u_{i}=\tau_{i}$. The state space of the *i*th subsystem is

(5) $$\left\{\begin{array}{c} {\dot{x}}_{i1}=x_{i2}\\ {\dot{x}}_{i2}=f_{i}\left(x\right)+g_{i}u_{i} \end{array}\right.,$$

where

(6) $$\begin{array}{c} g_{i}={\left(I_{im}\gamma_{i}\right)}^{-1}\\ f_{i}=g_{i}\left(\begin{array}{c} -\left({\hat{f}}_{is}e^{\left(-{\hat{f}}_{ir}{\dot{x}}_{i1}^{2}\right)}+{\hat{f}}_{ic}\right)sgn\left(x_{i2}\right)-f_{ip}\left(x_{i1},x_{i2}\right)-\\ {\hat{f}}_{ib}x_{i2}-Y_{i}\left(x_{i2}\right){\tilde{F}}_{ir}-\frac{\tau_{is}}{\gamma_{i}}-I_{i}\left(x,\dot{x},\ddot{x}\right)-J_{i}^{T}f \end{array}\right). \end{array}$$

Control objectives aim to ensure optimal tracking error performance for the MUS in coordinate control. Within the subsequent section, we introduce an event-trigger reinforcement learning-based coordinate control via nonzero-sum game framework.

## 4. Event-Trigger Reinforcement Learning-Based Coordinate Control via Nonzero-Sum Game

### 4.1. Problem Transformation

Based on the dynamic model (1) and state space (5), the control object of this paper is completing optimal trajectory tracking. Therefore, to facilitate designing the controller, the augmenting subsystem is deduced:

(7) $$\left\{\begin{array}{c} {\dot{x}}_{1}=x_{2}\\ {\dot{x}}_{2}=f\left(x\right)+\sum_{m=1}^{n}G_{m}u_{m} \end{array}\right.,$$

where $x={\left[x_{1}^{T},x_{2}^{T}\right]}^{T}\in R^{2n}$ is global state of the MUS, in which the vectors $x_{1},x_{2}$ are given by $x_{1}=[x_{11},\cdots ,x_{i1},\cdots ,x_{n1}{]}^{T}\in R^{n}$ and $x_{2}={\left[x_{12},\cdots ,x_{i2},\cdots ,x_{n2}\right]}^{T}\in R^{n}$. Moreover, $f\left(x\right)=[f_{1}\left(x\right),\cdots ,f_{i}\left(x\right),\cdots ,f_{n}\left(x\right){]}^{T}$, $G_{m}={\left[0,\cdots ,0,g_{m},0,\cdots ,0\right]}^{T}$, where $g_{m}={\left(I_{mm}\gamma_{m}\right)}^{-1},m=1,\cdots ,n$.

Define the cost function:

(8) $$\begin{array}{cc} J_{i}\left({\dot{e}}_{s},u_{1},\cdots ,u_{n}\right) & =\int_{t}^{\infty }\left({{\dot{e}}^{T}}_{s}Q_{i}{\dot{e}}_{s}+\sum_{m=1}^{n}u_{m}^{T}R_{im}u_{m}\right)d\tau \\ \; & =\int_{t}^{\infty }U_{i}\left({\dot{e}}_{s},u_{1},\cdots ,u_{n}\right)d\tau , \end{array}$$

where position error is $e={\left[e_{1},e_{2},\cdots ,e_{n}\right]}^{T}=x_{1}-x_{d}$ and velocity error vector $\dot{e}={\left[{\dot{e}}_{1},{\dot{e}}_{2},\cdots ,{\dot{e}}_{n}\right]}^{T}=x_{2}-{\dot{x}}_{d}$; $e_{s}=\dot{e}+\beta e$ means fusion error; $x_{d},{\dot{x}}_{d},{\ddot{x}}_{d}$ represent the determined reference vectors; $Q_{i},R_{im}$ denote determined positive definite matrices; and $U_{i}\left({\dot{e}}_{s},u_{1},\cdots ,u_{n}\right)$ indicates the utility function. Employing the infinitesimal version of (8), the Hamiltonian function can be derived:

(9) $$H_{i}\left({\dot{e}}_{s},u_{1},\cdots ,u_{n},\nabla J_{i}\right)=U_{i}\left({\dot{e}}_{s},u_{1},\cdots ,u_{n}\right)+{\left(\nabla J_{i}\right)}^{T}\left(f\left(x\right)+\sum_{m=1}^{n}G_{m}u_{m}-b_{d}\right),$$

where $\nabla J_{i}\left({\dot{e}}_{s}\right)=\frac{\partial J_{i}\left({\dot{e}}_{s}\right)}{\partial {\dot{e}}_{s}}$ is the partial derivative of $J_{i}\left({\dot{e}}_{s}\right)$, $b_{d}={\ddot{x}}_{d}+\beta e$. Additionally, the optimal value function can be described as

(10) $$J_{i}^{*}\left({\dot{e}}_{s},u_{1},\cdots ,u_{n}\right)=\min_{u_{i}}\;\int_{t}^{\infty }U_{i}\left({\dot{e}}_{s},u_{1},\cdots ,u_{n}\right)d\tau .$$

Based on the stationary condition $\frac{\partial H_{i}}{\partial u_{i}}=0$, the local optimal control policy $u_{i}^{*}$ is defined as

(11) $$u_{i}^{*}=-\frac{1}{2}R_{ii}^{-1}G_{i}^{T}\nabla J_{i}^{*}.$$

By substituting (8) and (11) into the Hamiltonian function (9), the coupled Hamilton–Jacobi (HJ) equation can be derived:

(12) $$\begin{array}{cc} 0 & ={\left(\nabla J_{i}^{*}\right)}^{T}\left(\;\;f\left(x\right)-\frac{1}{2}\sum_{m=1}^{n}G_{m}R_{mm}^{-1}G_{m}^{T}\nabla J_{m}^{*}+\varpi \left(x\right)-b_{d}\right)\\ \; & +\frac{1}{4}\sum_{m=1}^{n}{\left(\nabla J_{i}^{*}\right)}^{T}G_{m}R_{mm}^{-1}R_{im}R_{mm}^{-1}G_{m}^{T}\left(\nabla J_{m}^{*}\right)+{{\dot{e}}^{T}}_{s}Q_{i}{\dot{e}}_{s} \end{array}$$

It is hard to obtain an analytical solution because of the nonlinearity system. Therefore, an event-trigger reinforcement learning-based coordinate control is introduced.

### 4.2. Event-Trigger Reinforcement Learning-Based Coordinate Control

The optimal control policy (11) is addressed from periodic sampling as well as the coupled HJ equation (12). Fixed sampling control not only escalates computational demands but also excessively taps into communication resources, jeopardizing the timeliness of control in environments with constrained bandwidth. Therefore, an event-triggered strategy is introduced to optimize efficiency.

Set a series of monotonously increasing ${\left\{t_{j}\right\}}_{j=0}^{+\infty }$, which contains trigger instants $t_{j}$. Then, define the sampling state

(13) $${\dot{e}}_{sji}\left(x_{ji}\right)={\dot{e}}_{sji}\left(x_{i}\left(t_{j}\right)\right),$$

where ${\dot{e}}_{sji}\left(x_{ji}\right)$ denotes triggering instant state for $t\in \left[t_{j},t_{j+1}\right)$. To obtain the trigger condition, the subsequent gap function is introduced:

(14) $$g_{eji}\left(t\right)={\dot{e}}_{si}\left(x_{i}\right)-{\dot{e}}_{sji}\left(x_{i}\right).$$

Upon event triggering, based on (13), the actual state undergoes sampling to become the sampled state, after which $g_{eji}\left(t\right)$ is reset to zero. The optimal control law is updated to ${u_{i}}^{*}\left({\dot{e}}_{si}\left(t_{j}\right)\right)$$={u_{i}}^{*}\left({\dot{e}}_{sji}\right)$ during $\left[t_{j},t_{j+1}\right)$, j∈N. It should be noted that ${u_{i}}^{*}\left({\dot{e}}_{sji}\right)$ are discrete values updated irregularly, necessitating conversion to continuous values. Therefore, a zero-order holder is derived to cope with this issue.

According to the dynamic model of MUS (7), one gives the event-trigger value function as follows:

(15) $$J_{i}\left({\dot{e}}_{sji},u_{1},\cdots ,u_{n}\right)=\int_{t}^{t+T}\left({{\dot{e}}_{sji}}^{T}Q_{i}{\dot{e}}_{sji}+\sum_{m=1}^{n}u_{m}^{T}\left({\dot{e}}_{sji}\right)R_{im}u_{m}\left({\dot{e}}_{sji}\right)\right)d\tau .$$

One has the event-triggered HJ equation:

(16) $$\begin{array}{cc} \; & H_{i}\left({\dot{e}}_{sji},u_{1},\cdots ,u_{n},\nabla J_{i}\left({\dot{e}}_{sji}\right)\right)=U_{i}\left({\dot{e}}_{sji},u_{1},\cdots ,u_{n}\right)\\ \; & \;+{\left(\nabla J_{i}\left({\dot{e}}_{sji}\right)\right)}^{T}\left(f\left(x\right)+\sum_{m=1}^{n}G_{m}u_{m}\left({\dot{e}}_{sji}\right)-b_{d}\right), \end{array}$$

where $\nabla J_{i}\left({\dot{e}}_{sji}\right)=\partial J_{i}\left({\dot{e}}_{sji}\right)/\partial {\dot{e}}_{sji}$ is the partial derivative of $J_{i}\left({\dot{e}}_{sji}\right)$ with regard to ${\dot{e}}_{sji}$. To eliminate the assumption of norm-boundness regarding interconnections, the desired states of coupled subsystems are used as a replacement for their actual states. Consequently, the interconnection term is depicted:

(17) $$\begin{array}{cc} \; & f\left(x\right)=f_{i}\left(x_{i},x_{md}\right)+\Delta f_{i}\left(x,x_{md}\right),\\ \; & u_{m}=G_{m}^{-1}\left({\dot{x}}_{m2d}-f_{m}\left(x_{d}\right)\right),m\neq i. \end{array}$$

where $x_{md}$ is the desired state of coupled subsystems for m=1,⋯,i−1,i+1,⋯,n, and $\Delta f_{i}\left(x,x_{md}\right)$ represents the substitution error. Given the interconnection’s compliance with the global Lipschitz condition, this indicates

(18) $$∥\Delta f_{i}\left(x,x_{md}\right)∥\leq \sum_{m=1,m\neq i}^{n}d_{im}E_{m},$$

where $E_{m}=∥x_{m}-x_{md}∥,$ and $d_{im}\geq 0$ denotes an unknown global Lipschitz constant.

The improved event-triggered optimal value function is

(19) $$J_{i}^{*}\left({\dot{e}}_{sji},u_{1},\cdots ,u_{n}\right)\;=\;\min_{\mu_{i}}\left(\int_{t}^{t+T}\left({{\dot{e}}_{sji}}^{T}Q_{i}{\dot{e}}_{sji}+\sum_{m=1}^{n}u_{m}^{T}\left({\dot{e}}_{sji}\right)R_{im}u_{m}\left({\dot{e}}_{sji}\right)\right)d\tau \right).$$

Through the substitution of (19) into (16), it can be inferred that

(20) $$0=\min_{u_{i}\left({\dot{e}}_{sji}\right)\in \Psi_{i}\left(\Omega \right)}\;H_{i}\left({\dot{e}}_{sji},u_{i}\left({\dot{e}}_{sji}\right),\nabla {J_{i}}^{*}\left({\dot{e}}_{sji}\right)\right).$$

Based on (21), one has the event-triggered optimal control law

(21) $${u_{i}}^{*}\left({\dot{e}}_{sji}\right)=-\frac{1}{2}R_{ii}^{-1}G_{i}^{T}\nabla {J_{i}}^{*}\left({\dot{e}}_{sji}\right).$$

For any ${\dot{e}}_{si},\;{\dot{e}}_{sji}\in \Omega$, the control law is Lipschitz continuous. Then, one has a constant $\chi_{i}$ satisfying

(22) $$\begin{array}{cc} ∥{u_{i}}^{*}\left({\dot{e}}_{si}\right)-{u_{i}}^{*}\left({\dot{e}}_{sji}\right)∥ & =∥{u_{i}}^{*}\left({\dot{e}}_{si}+g_{eji}\left(t\right)\right)-{u_{i}}^{*}\left({\dot{e}}_{sji}\right)∥\leq \chi_{i}∥g_{eji}\left(t\right)∥. \end{array}$$

Given the challenging nature of solving the coupled HJ equation and the curse of dimensionality that arises with increasing dimensions, we employ the reinforcement learning algorithm for deriving an approximate solution for the event-triggered HJ equation in real-time.

The improved value function ${J_{i}}^{*}\left({\dot{e}}_{sji}\right)$ can be obtained by the radial basis function neural network (RBFNN) as follows:

(23) $${J_{i}}^{*}\left({\dot{e}}_{sji}\right)={W_{ci}}^{T}\delta_{ci}\left({\dot{e}}_{sji}\right)+\varepsilon_{ci}\left({\dot{e}}_{sji}\right),$$

where $W_{ci}\in R^{K_{i}}$ is the desired critic NN weight vector; $K_{i}$ is the number of neurons in the hidden-layer; $\delta_{ci}\left({\dot{e}}_{sji}\right)=\exp\left(-∥{\dot{e}}_{sji}-c_{ij}∥/2b_{ij}^{2}\right)$ denotes the activation function; and $\varepsilon_{ci}\left({\dot{e}}_{sji}\right)$ is critic NN approximation error, which is bounded as $∥\delta_{ci}\left({\dot{e}}_{sji}\right)∥$ $\leq \delta_{ci\;\max}$, $∥\varepsilon_{ci}\left({\dot{e}}_{sji}\right)∥\leq \varepsilon_{ci\;\max}$ with the positive constants $\delta_{ci\;\max}$ and $\varepsilon_{ci\;\max}$.

Therefore, the partial derivative of ${J_{i}}^{*}\left({\dot{e}}_{sji}\right)$ can be obtained as follows:

(24) $$\nabla {J_{i}}^{*}\left({\dot{e}}_{sji}\right)=\nabla \delta_{ci}^{T}\left({\dot{e}}_{sji}\right)W_{ci}+\nabla \varepsilon_{ci}\left({\dot{e}}_{sji}\right),$$

where $\nabla \delta_{ci}\left({\dot{e}}_{sji}\right)$ is Lipschitz continuous.

The relationship $∥\nabla \delta_{ci}\left({\dot{e}}_{si}\right)-\nabla \delta_{ci}\left({\dot{e}}_{sji}\right)∥\leq p_{i}∥g_{eji}\left(t\right)∥$ can be derived, and $p_{i}$ is a positive constant.

Substituting (24) into (21) can yield the following:

(25) $$\begin{array}{cc} {u_{i}}^{*}\left({\dot{e}}_{sji}\right) & =-\frac{1}{2}R_{ii}^{-1}G_{i}^{T}\nabla {J_{i}}^{*}\left({\dot{e}}_{sji}\right)\\ \; & =-\frac{1}{2}R_{ii}^{-1}G_{i}^{T}\left(\nabla \delta_{ci}^{T}\left({\dot{e}}_{sji}\right)W_{ci}+\nabla \varepsilon_{ci}\left({\dot{e}}_{sji}\right)\right). \end{array}$$

Therefore, one obtains the event-triggered HJ equation as follows:

(26) $$\begin{array}{c} H_{i}\left({\dot{e}}_{sji},u_{i}^{*}\left({\dot{e}}_{sji}\right),\nabla J_{i}^{*}\left({\dot{e}}_{sji}\right)\right)=U_{i}\left({\dot{e}}_{sji},u_{1},\cdots ,u_{i}^{*},\cdots ,u_{n}\right)\\ +{\left(\nabla J_{i}\left({\dot{e}}_{sji}\right)\right)}^{T}\left(\begin{array}{c} f_{i}\left(x_{i},x_{md}\right)-b_{d}+\sum_{m=1}^{n}G_{m}u_{m}\left({\dot{e}}_{sji}\right) \end{array}\right)≜e_{cHi}, \end{array}$$

where $e_{cHi}=-\nabla \varepsilon_{ci}^{T}\left({\dot{e}}_{sji}\right){\ddot{e}}_{sji}$ means residual error, and the positive constant $e_{cHi\;\max}$ is the upper bound of $e_{cHi}$.

Since we cannot obtain the desired critic NN weight vector, we approximate the improved value function

(27) $${\hat{J}}_{i}\left({\dot{e}}_{sji}\right)={\hat{W}}_{ci}^{T}\delta_{ci}\left({\dot{e}}_{sji}\right).$$

Furthermore, the partial derivative ${\hat{J}}_{i}\left({\dot{e}}_{sji}\right)$ is formulated as follows:

(28) $$\nabla {\hat{J}}_{i}\left({\dot{e}}_{sji}\right)=\nabla \delta_{ci}^{T}\left({\dot{e}}_{sji}\right){\hat{W}}_{ci}.$$

Therefore, merging (28) with (21), the event-trigger-based approximate optimal control law ${\hat{u}}_{i}\left({\dot{e}}_{sji}\right)$ is obtained as

(29) $${\hat{u}}_{i}\left({\dot{e}}_{sji}\right)=-\frac{1}{2}R_{ii}^{-1}G_{i}^{T}\left(\nabla \delta_{ci}^{T}\left({\dot{e}}_{sji}\right){\hat{W}}_{ci}\right).$$

According to (25), (28) and (29), we can obtain the event-triggered approximate HJ equation as

(30) $$\begin{array}{c} {\hat{H}}_{i}\left({\dot{e}}_{sji},{{\hat{u}}_{i}}^{*}\left({\dot{e}}_{sji}\right),\nabla {{\hat{J}}_{i}}^{*}\left({\dot{e}}_{sji}\right)\right)=U_{i}\left({\dot{e}}_{sji},{\hat{u}}_{1},\cdots ,{\hat{u}}_{i}^{*},\cdots ,{\hat{u}}_{n}\right)\\ +{\left(\nabla J_{i}\left({\dot{e}}_{sji}\right)\right)}^{T}\left(\begin{array}{c} f_{i}\left(x_{i},x_{md}\right)-b_{d}+\sum_{m=1}^{n}G_{m}{\hat{u}}_{m}\left({\dot{e}}_{sji}\right) \end{array}\right)≜e_{ci}. \end{array}$$

Define the critic approximation error vector as ${\tilde{W}}_{ci}=W_{ci}-{\hat{W}}_{ci}$; from (30), we can define $\partial e_{ci}/\partial {\hat{W}}_{ci}=\nabla \delta_{ci}\left({\dot{e}}_{sji}\right)$${\ddot{e}}_{sji}=\theta_{i}$. To refine the estimation of the desired vector ${\hat{W}}_{ci}$, we employ the gradient descent algorithm to minimize the objective function $E_{ci}=\frac{1}{2}e_{ci}^{2}$, with the update rate given by

(31) $$\begin{array}{cc} {\dot{\hat{W}}}_{ci} & =-\alpha_{ci}\frac{\partial E_{ci}}{\partial {\hat{W}}_{ci}}=-\alpha_{ci}\left(U_{i}\left({\dot{e}}_{sji},{\hat{u}}_{1},\cdots ,{\hat{u}}_{i}^{*},\cdots ,{\hat{u}}_{n}\right)+\theta_{i}{\hat{W}}_{ci}^{T}\right)\theta_{i}. \end{array}$$

Then, the approximation error vector is

(32) $${\dot{\tilde{W}}}_{ci}=-\alpha_{ci}\left(\theta_{i}{\tilde{W}}_{ci}^{T}-e_{cHi}\right)\theta_{i}.$$

**Theorem** **1.** *Taking the value function (23) into account, it is estimated by the critic NN with weights $W_{ci}$. The cost function, as given by Equation (27), is approximated using the weights ${\hat{W}}_{ci}$. Assuming the update law for the critic NN is defined by (31), the weight approximation error is proven to be UUB.*

**Proof.**  The candidate for the Lyapunov function is selected as

(33) $$V_{1i}\left(t\right)=\frac{1}{2\alpha_{ci}}{\tilde{W}}_{ci}^{T}{\tilde{W}}_{ci}.$$

The derivative of $V_{1i}\left(t\right)$ can be obtained as

(34) $$\begin{array}{c} {\dot{V}}_{1i}\left(t\right)=\frac{1}{\alpha_{ci}}{\tilde{W}}_{ci}^{T}{\dot{\tilde{W}}}_{ci}={\tilde{W}}_{ci}^{T}\left(e_{cHi}-\theta_{i}{\tilde{W}}_{ci}^{T}\right)\theta_{i}\\ ={\tilde{W}}_{ci}^{T}e_{cHi}\theta_{i}-{∥{\tilde{W}}_{ci}^{T}\theta_{i}∥}^{2}\leq \frac{1}{2}e_{cHi}^{2}-\frac{1}{2}{∥{\tilde{W}}_{ci}^{T}\theta_{i}∥}^{2}. \end{array}$$

Upon analyzing (34), we observe that if $∥{\tilde{W}}_{ci}∥\geq ∥\frac{e_{cHi}}{\theta_{i}}∥$, this leads to ${\dot{V}}_{1i}\left(t\right)<0$, which in turn confirms the UUB of the critic approximation error vector. □

**Theorem** **2.** *Given an MUS with joint subsystem dynamic model (1) and state space (7), the closed-loop MUS with coordinate control is UUB under the presented event-triggered reinforcement learning-based coordinate control law (35) if*

(35) $$\begin{array}{cc} {∥g_{eji}∥}^{2} & \leq \frac{\left(1\;-\;\alpha_{i}^{2}\right)\sigma_{\min}\left(Q_{i}\right){∥{\dot{e}}_{si}∥}^{2}\;\;+\;\;\sum_{m=1}^{n}{∥r_{m}∥}^{2}{∥{\hat{u}}_{m}\left({\dot{e}}_{sjm}\right)∥}^{2}}{2R_{i}\chi_{i}^{2}}\\ & -\frac{G_{i}^{2}{\left(\nabla \delta_{ci\;\max}W_{ci\;\max}+\nabla \varepsilon_{ci\;\max}\right)}^{2}}{4R_{i}^{2}\chi_{i}^{2}}, \end{array}$$

*holds, where $\alpha_{i}\in \left(0,1\right)$ is the designed sampling frequency parameter, $\sigma_{\min}\left(\cdot \right)$ means the minimum eigenvalue of the matrix, and $r_{im}$ is a positive constant that satisfies $R_{im}=r_{im}^{T}r_{im}$, assuming $∥{\tilde{W}}_{ci}∥\leq ∥W_{ci\;\max}∥$.*

**Proof.**  We select the Lyapunov candidate function

(36) $$V_{i}\left(t\right)=V_{si}+V_{sji},$$

where $V_{si}={J_{i}}^{*}\left({\dot{e}}_{si}\right)$ and $V_{sji}={J_{i}}^{*}\left({\dot{e}}_{sji}\right)$. □

The following proof is divided into two cases.

**Case 1:**  The events are not triggered, i.e., $t\in \left[t_{j},t_{j+1}\right)$.

Computing the derivative with respect to time of (36), the result is obtained as follows:

(37) $${\dot{V}}_{si}\left(t\right)={\left(\nabla J_{i}^{*}\left({\dot{e}}_{si}\right)\right)}^{T}\left(\begin{array}{c} f_{i}\left(x_{i},x_{md}\right)-b_{d}+\sum_{m=1}^{n}G_{m}u_{m} \end{array}\right),$$

(38) $${\dot{V}}_{sji}=0.$$

Based on the optimal control law (11) and time-triggered HJ equation (12), one has

(39) $$\begin{array}{cc} {\left(\nabla J_{i}^{*}\right)}^{T}\left(f_{i}\left(x_{i},x_{md}\right)-b_{d}\right)= & -{\dot{e}}_{si}^{T}Q_{i}{\dot{e}}_{si}+\frac{1}{2}\sum_{m=1}^{n}G_{m}R_{mm}^{-1}G_{m}^{T}\nabla J_{m}^{*}\\ \; & -\frac{1}{4}\sum_{m=1}^{n}{\left(\nabla J_{m}^{*}\right)}^{T}G_{m}R_{mm}^{-1}R_{im}R_{mm}^{-1}G_{m}^{T}\left(\nabla J_{m}^{*}\right), \end{array}$$

(40) $$\nabla {J_{i}}^{*}\left({\dot{e}}_{si}\right)G_{i}=-2\sum_{m=1}^{n}G_{m}^{T}u_{m}^{*}.$$

Substituting (39) and (40) into (37), we can obtain the following equation:

(41) $$\begin{array}{cc} {\dot{V}}_{si}\left(t\right) & =-{\dot{e}}_{si}^{T}Q_{i}{\dot{e}}_{si}-{\left(\nabla J_{i}^{*}\right)}^{T}\left(\sum_{m=1}^{n}G_{m}\left(u_{m}^{*}-{\hat{u}}_{m}\right)\right)-\frac{1}{4}\sum_{m=1}^{n}\left({\left(\nabla J_{m}^{*}\right)}^{T}G_{m}R_{mm}^{-1}R_{im}R_{mm}^{-1}G_{m}^{T}\left(\nabla J_{m}^{*}\right)\right)\\ \; & =-{\dot{e}}_{si}^{T}Q_{i}{\dot{e}}_{si}-\frac{1}{4}\sum_{m=1}^{n}\;\;\left(\begin{array}{c} {\left(\nabla J_{m}^{*}\left({\dot{e}}_{sji}\right)\right)}^{T}G_{m}R_{mm}^{-1}R_{im}R_{mm}^{-1}G_{m}^{T}\left(\nabla J_{m}^{*}\left({\dot{e}}_{sji}\right)\right) \end{array}\right)\\ \; & +\frac{1}{2}{\left(\begin{array}{c} \nabla \delta_{ci}^{T}\left({\dot{e}}_{sji}\right)W_{ci}+\nabla \varepsilon_{ci} \end{array}\right)}^{T}\left(\sum_{m=1}^{n}G_{m}R_{mm}^{-1}\left(\begin{array}{c} G_{m}^{T}\nabla \delta_{cm}^{T}\left({\dot{e}}_{sji}\right){\tilde{W}}_{cm}+G_{m}^{T}\nabla \varepsilon_{cm} \end{array}\right)\right)\\ \; & =-{\dot{e}}_{si}^{T}Q_{i}{\dot{e}}_{si}-\frac{1}{4}\sum_{m=1}^{n}\left(\begin{array}{c} {\left(\nabla J_{m}^{*}\left({\dot{e}}_{sji}\right)\right)}^{T}G_{m}R_{mm}^{-1}R_{im}R_{mm}^{-1}G_{m}^{T}\left(\nabla J_{m}^{*}\left({\dot{e}}_{sji}\right)\right) \end{array}\right)+\Pi_{iJ}, \end{array}$$

in which the function term $\Pi_{iJ}$ has the following up-bound:

(42) $$\begin{array}{cc} \Pi_{iJ} & \leq ∥\begin{array}{c} \frac{1}{2}{\left(\nabla \delta_{ci}^{T}\left({\dot{e}}_{sji}\right)W_{ci}+\nabla \varepsilon_{ci}\right)}^{T}\left(\sum_{m=1}^{n}G_{m}R_{mm}^{-1}G_{m}^{T}\left(\nabla \phi_{cm}^{T}\left({\dot{e}}_{sji}\right){\tilde{W}}_{cm}+\nabla \varepsilon_{cm}\right)\right) \end{array}∥\leq \pi_{iJ}, \end{array}$$

where $\pi_{iJ}$ is a computable positive constant.

According to (24) and (28), (41) can be transformed into

(43) $${\dot{V}}_{si}\leq -{{\dot{e}}_{si}}^{T}Q_{i}{\dot{e}}_{si}-\sum_{m=1}^{n}{{\hat{u}}_{m}}^{T}\left({\dot{e}}_{sjm}\right)R_{im}{\hat{u}}_{m}\left({\dot{e}}_{sjm}\right)+2\chi^{2}R_{ii}{∥g_{eji}\left(t\right)∥}^{2}\;\;+\;\;\frac{1}{2}R_{ii}^{-1}G_{i}^{2}{\left(\nabla \delta_{ci}^{T}\left({\dot{e}}_{sji}\right){\tilde{W}}_{ci}\;\;+\;\;\nabla \varepsilon_{ci}\left({\dot{e}}_{sji}\right)\right)}^{2}.$$

Thus, we have $\dot{V}\left(t\right)$ as

(44) $$\dot{V}\left(t\right)=\sum_{i=1}^{n}{\dot{V}}_{i}\left(t\right)=\sum_{i=1}^{n}\left({\dot{V}}_{si}+{\dot{V}}_{sji}\right).$$

According to (38) and (43) as well as (44), one has

(45) $$\begin{array}{cc} \dot{V}\left(t\right) & \leq \sum_{i=1}^{n}\left(\begin{array}{c} -{{\dot{e}}_{si}}^{T}Q_{i}{\dot{e}}_{si}+2R_{im}\chi^{2}{∥g_{eji}∥}^{2}-\sum_{m=1}^{n}{{\hat{u}}_{m}}^{T}\left({\dot{e}}_{sjm}\right)R_{im}{\hat{u}}_{m}\left({\dot{e}}_{sjm}\right)\\ +\frac{1}{2}R_{im}^{-1}G_{i}^{2}{\left(\nabla \delta_{ci}^{T}\left({\dot{e}}_{sji}\right){\tilde{W}}_{ci}+\nabla \varepsilon_{ci}\left({\dot{e}}_{sji}\right)\right)}^{2} \end{array}\right)\\ \; & \leq \sum_{i=1}^{n}\left(\begin{array}{c} -\alpha_{i}^{2}\sigma_{\min}\left(Q_{i}\right){∥{\dot{e}}_{si}∥}^{2}+\left(\alpha_{i}^{2}-1\right)\sigma_{\min}\left(Q_{i}\right){∥{\dot{e}}_{si}∥}^{2}\;-\;{∥r_{im}∥}^{2}{∥{\hat{u}}_{i}\left({\dot{e}}_{sjm}\right)∥}^{2}\\ +2R_{im}\chi^{2}{∥g_{eji}∥}^{2}+\frac{1}{2}R_{im}^{-1}G_{i}^{2}{\left(\nabla \delta_{ci\;\max}W_{ci\;\max}+\nabla \varepsilon_{ci\;\max}\right)}^{2} \end{array}\right). \end{array}$$

Given that (35) is valid, (45) is compliant with the requirement $\dot{V}\left(t\right)\leq \sum_{i=1}^{n}(\pi_{iJ}-\alpha_{i}^{2}\sigma_{\min}\left(Q_{i}\right){∥{\dot{e}}_{si}∥}^{2})$. The condition that ensures the negativity of ${\dot{V}}_{i}\left(t\right)$ is that ${\dot{e}}_{si}$ does not fall within the confines of $\Omega \;=\;\left\{{\dot{e}}_{si}:∥{\dot{e}}_{si}∥\;\leq \;\sqrt{\frac{\pi_{iJ}}{\alpha_{i}^{2}\sigma_{\min}\left(Q_{i}\right)}}\right\}$, a requirement critical for affirming the negativity of the proposed Lyapunov function.

**Case 2:**  When events are triggered, $\forall t=t_{j+1}$, the difference of (36) is rewritten as
  (46) $$\begin{array}{cc} \Delta V_{i}\left(t\right) & =V_{i}\left({\dot{e}}_{sji+1}\right)-V_{i}\left({\dot{e}}_{si}\left(x_{ji+1}^{-}\right)\right)\\ \; & =J_{i}^{*}\left({\dot{e}}_{sji+1}\right)-J_{i}^{*}\left({\dot{e}}_{si}\left(x_{ji+1}^{-}\right)\right)+J_{i}^{*}\left({\dot{e}}_{sji+1}\right)-J_{i}^{*}\left({\dot{e}}_{sji}\right). \end{array}$$

Based on (35), one has $\dot{V}\left(t\right)\leq 0$. Therefore, we have ${J_{i}}^{*}\left({\dot{e}}_{sji+1}\right)$$\leq {J_{i}}^{*}\left({\dot{e}}_{si}\left(x_{ji+1}^{-}\right)\right)$.

Then, one has (46) as the following form:

(47) $$\Delta V\left(t\right)\leq {J_{i}}^{*}\left({\dot{e}}_{sji+1}\right)-{J_{i}}^{*}\left({\dot{e}}_{sji}\right)\leq -\nu ∥g_{eji}\left(t_{j}\right)∥,$$

where ν denotes a class-*k* function, $g_{eji}\left(t_{j}\right)={\dot{e}}_{sji+1}-{\dot{e}}_{sji}$.

Taking into account Cases 1 and 2 collectively, it follows that under the condition specified by (35), the closed-loop MUS’s tracking error is UUB. Thus, the conclusion of the proof is established.

### 4.3. Exclusion of Zeno Behaviors

The minimum trigger interval $t_{\min}=\min\left\{t_{j+1}-t_{j}\right\}$ is likely to be 0—that is, Zeno behavior. Therefore, we give the following theorem to avoid the phenomenon:

**Theorem** **3.** *Considering MUS (1), the triggering condition (35) and the event-triggered approximate optimal control law (29), the minimum trigger interval $t_{\min}$ is with a positive lower bound by*

(48) $$t_{\min}\geq \frac{1}{S_{iZ}}\ln\left(1+\pi_{j,\min}\right)>0,$$

*where $\pi_{j,\min}=\min\left(\frac{∥g_{eji}\left({\dot{e}}_{si}\left(x_{ji+1}^{-}\right)\right)∥}{\left({\dot{e}}_{sji}\right)+\Theta_{i}}\right)>0$, $S_{iZ},\Theta_{i}$ are positive constants.*

**Proof.**  The time derivative of the event-triggered gap function (14) can be derived as follows:

(49) $$\frac{d\left(g_{eji}\right)}{dt}={\dot{g}}_{eji}={\ddot{e}}_{si}\left(x_{i}\right)-{\ddot{e}}_{sji}\left(x_{ji}\right)={\ddot{e}}_{si}\left(x_{i}\right).$$

The upper bound of ${\ddot{e}}_{si}\left(x_{i}\right)$ is derived as

(50) $$∥{\ddot{e}}_{si}\left(x_{i}\right)∥\leq S_{iZ}∥x_{i}∥+S_{iZ}\Theta_{i}.$$

Combining (14) and (49) with (50), it can be obtained that

(51) $$\begin{array}{cc} ∥{\dot{g}}_{eji}∥ & \leq \int_{t_{j}}^{t}\left(e^{\left(S_{iZ}\left(t-w\right)\right)}S_{iZ}\left(∥{\dot{e}}_{sji}∥+\Theta_{i}\right)\right)dw\leq \left(∥{\dot{e}}_{sji}∥+\Theta_{i}\right)\left(e^{\left(S_{iZ}\left(t-t_{j}\right)\right)}-1\right). \end{array}$$

When $t=t_{j+1}$, the event-triggered condition satisfies

(52) $$∥g_{eji}\left({\dot{e}}_{sji}\left(x_{ji+1}^{-}\right)\right)∥=∥g_{eji}\left(x_{ji+1}^{-}\right)∥.$$

Based on (51) and (52), the *j*th triggering interval $\Delta t_{j}$ has the lower bound by

(53) $$\Delta t_{j}=t_{j+1}-t_{j}\geq \frac{1}{S_{iZ}}\ln\left(1+\frac{∥g_{eji}\left({\dot{e}}_{sji}\left(x_{ji+1}^{-}\right)\right)∥}{\left({\dot{e}}_{sji}\right)+\Theta_{i}}\right).$$

This concludes the proof. □

## 5. Experiment

### 5.1. Experimental Setup

**Figure 1:**
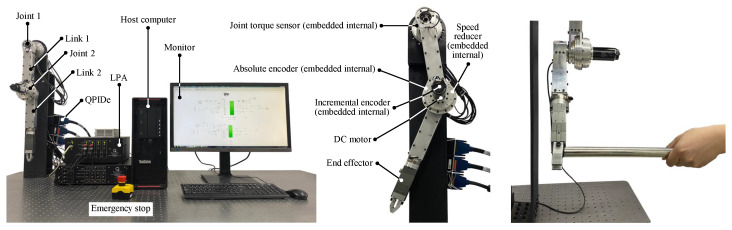
Experimental platform.

The validation of the proposed control method’s effectiveness is demonstrated through experiments on a 2 degrees of freedom (DOF) MUS platform. Detailed information about the experimental setup can be found in Figure 1. Joint control torque is measured using a joint torque sensor, while joint position information is acquired from both absolute and incremental encoders. The data acquisition board acts as the intermediary allowing interaction between the software environment (Simulink of Matlab 2016a) and hardware components. It is noted that the proposed ETRL via NZSG, which is in the form of continuous time, needs to be realized discretely when it is implemented in experiments. Fortunately, the control system, which is constructed under the Simulink environment, may complete the discrete realization automatically and adjust the sampling period adaptively. The model parameters are as follows: $I_{mi}$ = 120 g·cm^2^, $\gamma_{i}$ = 100, ${\hat{f}}_{ib}$ = 12 m·Nm/rad, ${\hat{f}}_{ic}$ = 30 m·Nm, ${\hat{f}}_{is}$ = 40 m·Nm, ${\hat{f}}_{i\tau }$ = 20 s^2^/rad^2^. We consider the coordinate control, which is illustrated in Figure 1. The purpose of the experiment is satisfying the requirements of position tracking performance and control torque optimization under a coordinate operation with MUS. The critic NN is selected as RBFNN, and the activation function of (23) is $\delta_{ci}=e^{\frac{-{\left({\dot{e}}_{sji}-\ell_{i}\right)}^{T}\left({\dot{e}}_{sji}-\ell_{i}\right)}{\zeta_{i}}}$ with initial value $W_{ci}={\left[0.3,0.3,0.3,0.3,0.3\right]}^{T}.$ $\ell_{i},\zeta_{i}$ denote the center and width of the activation function. The purpose of the control method is to decrease the position error and control torque as much as possible. The experimental results show that the proposed method reduces the position error by 30% and control torque by 10% compared with the existing control methods.

### 5.2. Experimental Results

The experimental outcomes are utilized to evaluate the system’s position tracking accuracy, tracking error magnitude, applied control torque, contact forces, event-triggering mechanism’s efficiency, and neural network (NN) weights’ performance individually. Two distinct control methodologies are implemented: the established learning-based tracking approach, as seen in references [37,39], and the novel proposed control strategy. The coordinate control was subjected to the implementation of two distinct control approaches. The upper figure corresponds to joint one, while the lower one illustrates joint two.

(1) Position tracking

**Figure 2:**
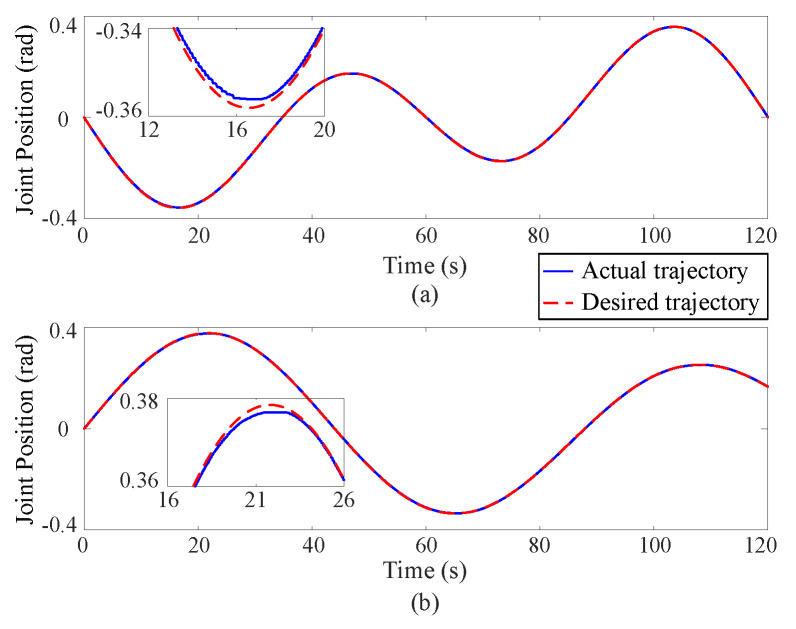
Position tracking curves in joint space via the existing learning-based tracking control method, where the upper (**a**) and lower (**b**) subgraphs correspond to Joint 1 and Joint 2 respectively.

**Figure 3:**
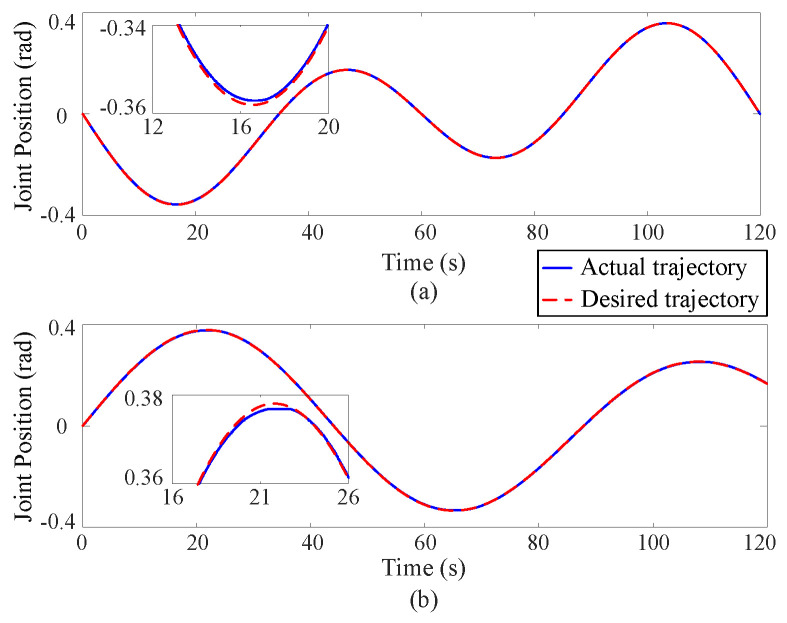
Position tracking curves in joint space via the proposed approximate optimal control method, where the upper (**a**) and lower (**b**) subgraphs correspond to Joint 1 and Joint 2 respectively.

**Figure 4:**
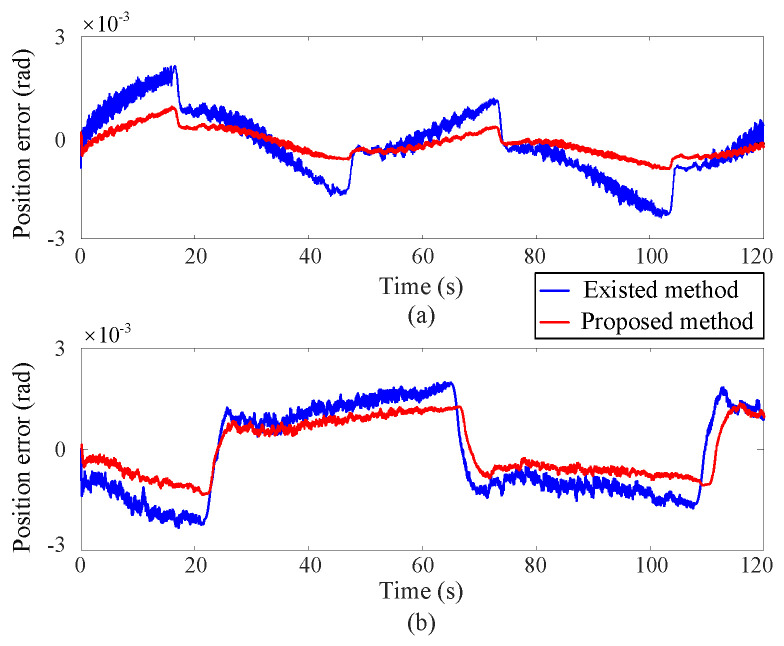
Position tracking error curves in joint space, where the upper (**a**) and lower (**b**) subgraphs correspond to Joint 1 and Joint 2 respectively.

**Figure 5:**
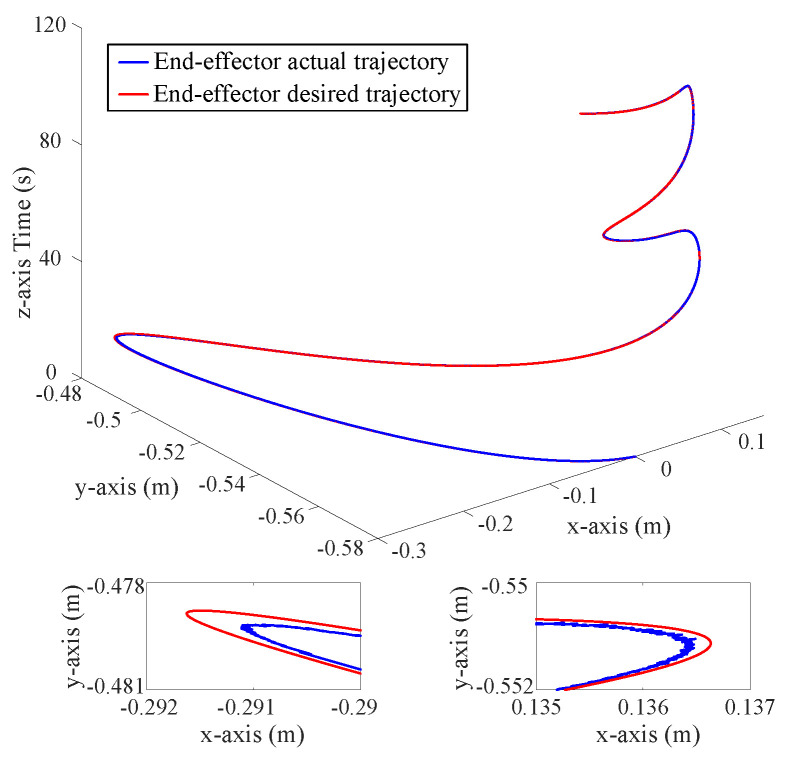
Position tracking curves in 3D space.

Figure 2, Figure 3 and Figure 4 depict the position tracking and tracking error curves in joint space during coordinate control using both the existing learning-based tracking control method and the proposed approximate optimal control method. The graphical analysis indicates that the position tracking error is notably lower and smoother with the newly proposed control method as opposed to the previously established method. The proposed method reduces the position error by 30%. This is attributed to the accurate solution of the coordinate control problem achieved by the proposed method. At the corners of the trajectory, the tracking error tends to increase but is effectively mitigated back to an acceptable range along the smooth path by the proposed approximate optimal control method. Figure 5 shows the 3D tracking curves.

(2) Control torque

**Figure 6:**
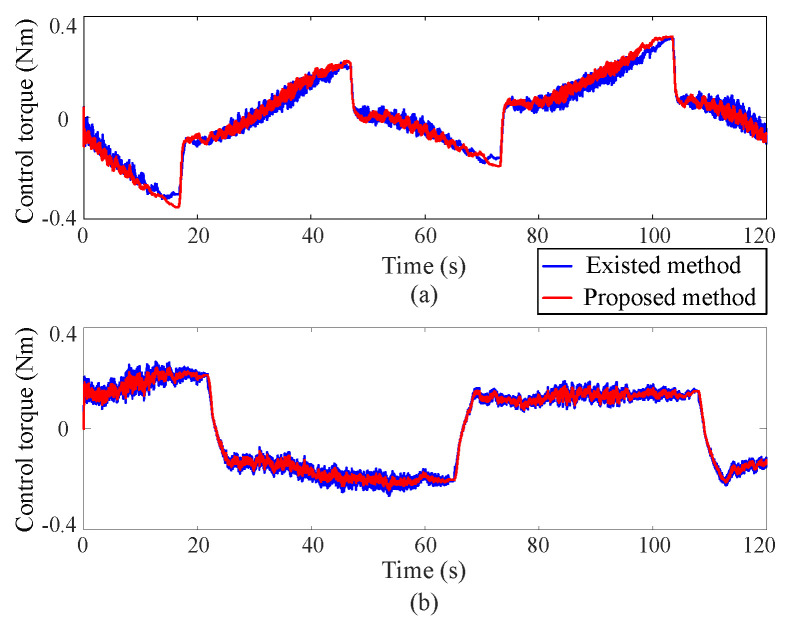
Control torque curves, where the upper (**a**) and lower (**b**) subgraphs correspond to Joint 1 and Joint 2 respectively.

Figure 6 displays the control torque curves during coordinate control using both the existing learning-based tracking control method and the proposed approximate optimal control method. The illustrations indicate that the control torque experiences a sharp increase during sudden trajectory changes, potentially impacting the lifespan of the DC motors. The proposed method reduces the control torque by 10% compared with the existing control methods. Furthermore, the control torque curves under the current control method display pronounced chattering, potentially degrading the accuracy of trajectory tracking. However, by employing the developed approximate optimal control method, the output torques are optimized to minimize motor power consumption and instantaneous increases in control torques are maintained within safe boundaries.

(3) Contact force

**Figure 7:**
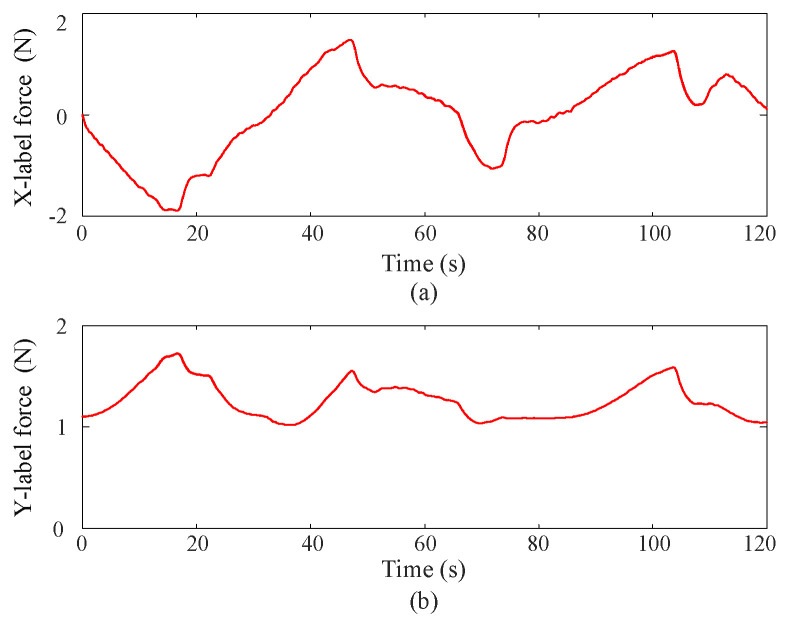
Contact force curves via the proposed approximate optimal control method, where the upper (**a**) and lower (**b**) subgraphs correspond to Joint 1 and Joint 2 respectively.

Figure 7 depicts the contact force curves during coordinate control using the proposed approximate optimal control method. Since the MUS has 2-DOF and the joint axes are assembled in parallel, the contact force curves appear in a two-dimensional space. From the figures, it can be observed that the proposed approximate optimal control method ensures that the contact force remains below 2N, with minimal chattering phenomenon.

(4) Event-triggered mechanism

**Figure 8:**
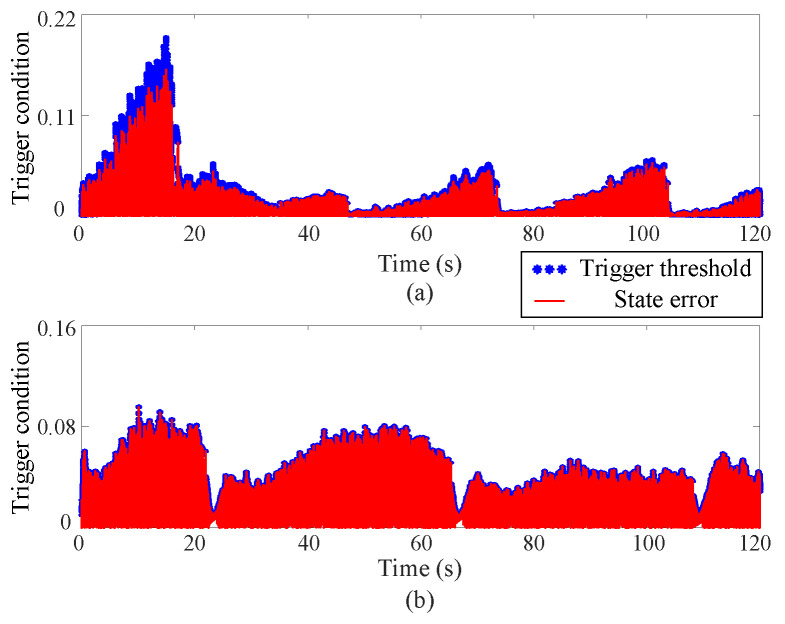
Trigger threshold and trigger condition curves via the proposed approximate optimal control method, where the upper (**a**) and lower (**b**) subgraphs correspond to Joint 1 and Joint 2 respectively.

**Figure 9:**
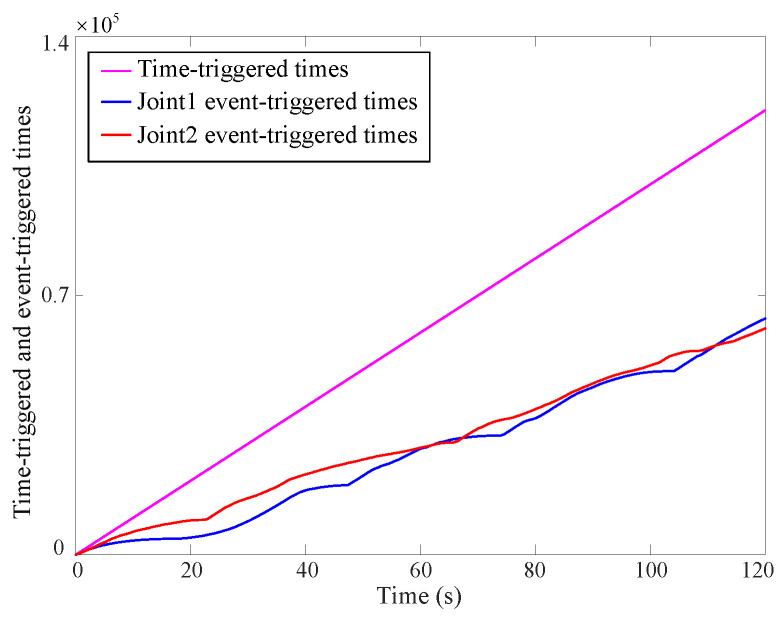
Time-triggered and event-triggered time curves via the proposed approximate optimal control method.

Figure 8 and Figure 9 depict the trigger threshold and trigger condition curves. Owing to the incorporation of the NN within the reinforcement learning process, both the trigger condition and the trigger threshold exhibit large values. The proposed method’s trigger time is nearly half that of the existing method. However, the trigger condition stays within the threshold limits, confirming the reliability of the newly introduced strategy. Figure 9 demonstrates that the developed controller substantially reduces the communication burden of the MUS.

(5) NN weight

**Figure 10:**
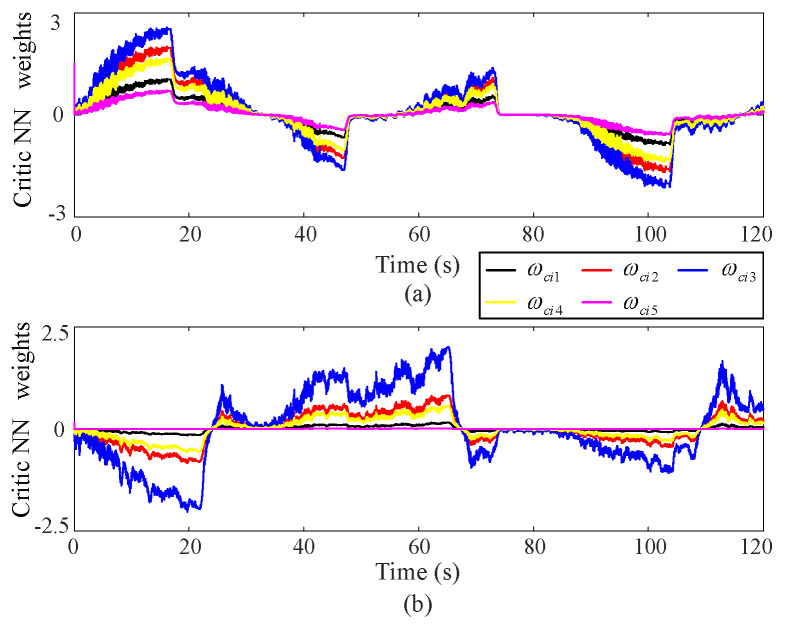
NN curve via the proposed approximate optimal control method, where the upper (**a**) and lower (**b**) subgraphs correspond to Joint 1 and Joint 2 respectively.

Figure 10 illustrates the behavior of the critic NN via RBFNN under coordinate control facilitated by the proposed approximate optimal control method. The converged weights obtained from the proposed approximate optimal control policies allow the NN to accurately reflect the ongoing coordinate operations in real-time.

Based on the experimental results, the closed-loop MUS systems have better performance than the existing methods in terms of position tracking and control torque under the proposed ETRL via the NZSG approach (cf. Table 1). Drawing from the experimental figure findings, when compared to existing methods, the closed-loop MUS demonstrates enhanced performance in position tracking, control torque, contact force, and event-triggered conditions under the proposed approximate optimal control method.

## 6. Conclusions

An ETRL-based coordinate control of MUS via NZSG is proposed in the paper. JTF is utilized to form the MUS’s dynamic. The coordinate control problem is transformed into an RL issue via the NZSG strategy. Conventional periodic communication is avoided by the ET mechanism. The performance index function is approximated by the critic NN to obtain the optimal control strategy. According to the Lyapunov theorem, the closed-loop system is guaranteed to be stable. The experimental results show that the proposed method reduces the position error by 30% and control torque by 10% compared with the existing control methods. The mentioned control algorithm only concerns the static event-trigger. However, the computation burden and power consumption can be optimized by the dynamic event-trigger or self-event-trigger. This is the future research direction that we will work on.

## Figures and Tables

**Table 1:** Performance comparisons.

	Mean Absolute Value of Position Error	Mean Absolute Value of Control Torque
The existing method (Joint 1)	$1.73\times 10^{-3}$ rad	0.32 Nm
The proposed method (Joint 1)	$1.03\times 10^{-3}$ rad	0.29 Nm
The existing method (Joint 2)	$1.62\times 10^{-3}$ rad	0.30 Nm
The existing method (Joint 2)	$0.98\times 10^{-3}$ rad	0.26 Nm

## Data Availability

The datasets generated during and/or analyzed during the current study are available from the corresponding author on reasonable request.
